# Antimicrobial Coating Based on Tahiti Lemon Essential Oil and Green Banana Flour to Preserve the Internal Quality of Quail Eggs

**DOI:** 10.3390/ani13132123

**Published:** 2023-06-27

**Authors:** Gabriel da Silva Oliveira, Concepta McManus, Cristiane Batista Salgado, Paula Gabriela da Silva Pires, Heloisa Alves de Figueiredo Sousa, Edilsa Rosa da Silva, Vinícius Machado dos Santos

**Affiliations:** 1 Faculty of Agronomy and Veterinary Medicine, University of Brasília, Brasília 70910-900, Brazil; gabriels.unb@gmail.com (G.d.S.O.);; 2 Laboratory of Geosciences and Human Sciences, Federal Institute of Brasília—Campus Brasília, Brasília 70830-450, Brazil; 3 Advanced Poultry Gut Science, Florianópolis 88000-000, Brazil; 4 Laboratory of Microbiology and Food, Federal Institute of Brasília—Campus Planaltina, Brasília 73380-900, Brazil; 5 Laboratory of Poultry Science, Federal Institute of Brasília—Campus Planaltina, Brasília 73380-900, Brazil

**Keywords:** coating, egg quality, egg storage, essential oils, microbiological quality

## Abstract

**Simple Summary:**

Coatings are possible alternatives to refrigeration that could be used to preserve quail eggs. These preservation alternatives arise after concerns, mainly because high temperatures and microorganisms negatively influence the maintenance of the internal quality of quail eggs, making them unfeasible for faster consumption. Thus, the aim of this study was to evaluate the microbiological and internal quality of quail eggs stored for 21 days at room temperature (29.53 ± 1.36 °C) after being coated with an antimicrobial coating based on green banana flour and Tahiti lemon essential oil (GBF/TAH).

**Abstract:**

This study evaluated the microbiological and internal quality of quail eggs stored for 21 days at room temperature (29.53 ± 1.36 °C) after being coated with green banana flour and Tahiti lemon essential oil (GBF/TAH). One hundred and sixty-two quail eggs were equally distributed into three treatments: (1) uncoated eggs, (2) eggs coated with green banana flour (GBF), and (3) eggs coated with GBF/TAH. The Haugh unit (HU) of the eggs was significantly lower in the third week for uncoated eggs (70.94 ± 1.63, grade A) compared to eggs coated with GBF/TAH (81.47 ± 2.38, grade AA). On the 21st day of storage, the eggs coated with GBF/TAH had significantly lower total counts of aerobic mesophilic bacteria in the shell and egg contents compared to the other treatments. GBF/TAH coating is an effective blending approach to reduce the microbial load of the shell and egg contents and preserve the sensory and internal quality of the eggs.

## 1. Introduction

Worldwide, approximately 10% of all eggs consumed are quail eggs [1]. A newly laid quail egg gradually becomes unfeasible for consumption as the deterioration process progresses. The closer to room temperature an egg is maintained, the faster it will degrade [2]. Initial and significant signs of deterioration in post-laying Japanese quail eggs at 19 °C were observed from 5 days after storage, in terms of weight, and after 3 days of storage, in terms of yolk and albumen quality [3]; meanwhile, they maintained excellent quality for up to 120 days when stored at 4 °C [4]. The increase in air temperature can also favor bacterial replication and the formation of bacterial biofilm on the surface of the eggshell, as well as internal contamination of the eggs, which significantly contributes to its degradation [5,6]. Among the microorganisms found in eggs that can be harmful to them are *Escherichia coli* and *Staphylococcus aureus* [7,8,9]. Due to the negligence with egg refrigeration in the industry and at points of sale in countries like Brazil [10], mainly due to the high demand for energy, as well as sophisticated facilities, accessible, ecological, and safe strategies have been developed to extend the shelf life of eggs, such as edible coatings. 

Coatings applicable to eggs are formed by the complex union of chemical molecules and play a significant role in preserving eggs during processing and marketing to ensure better quality eggs and a longer shelf life. This leads to less wastage of eggs, a reduction of economic losses, and consumers being prevented from ingesting eggs contaminated by pathogenic microorganisms, especially in cases where refrigeration is not adopted [11]. In terms of options, chitosan-coated quail eggs showed a better internal quality than uncoated eggs after 4 weeks of storage at 24 ± 2 °C [12]. Akpinar et al. [13] observed that coating quail eggs with propolis extract can help to decrease the loss of internal egg quality during storage. Renewable materials are environmentally friendly and hold promise in developing egg coatings. Despite the availability of different raw materials for the preparation of edible coatings, fruits produced on a large scale and that are available in several countries, such as bananas, deserve to be highlighted [14]. Salazar et al. [15] verified that green banana flour (GBF) may be a good alternative for industrial applications due to its ability to form filmogenic solutions, which are associated with the presence of starch in its composition [16]. GBF can be obtained both from the peel and pulp of a banana (*Musa* spp.) by drying [17]. 

Essential oils also make up a repertoire of bioavailable plant substances with antimicrobial, hydrophobic, and safe characteristics that have multiple laboratory and industrial applications. The delivery of essential oils in the formulation of coatings for eggs proposes some behaviors that are able to delay the deterioration of eggs, including the ability to control gaseous and water exchanges via the shell and improve the microbiological profile of the egg [18,19,20,21]. Tahiti lemon essential oil (*Citrus aurantifolia*; Rutaceae family) is an aggregate of volatile and non-volatile substances extracted mainly by cold pressing, which, when applied in cassava starch-based coating, preserved the internal quality and reduced total aerobic mesophilic bacteria in chicken eggshells [20]. Thus, the aim of this study was to evaluate the microbiological and internal quality of quail eggs stored for 21 days at room temperature (29.53 ± 1.36 °C) after being coated with an antimicrobial coating based on GBF and Tahiti lemon essential oil. 

## 2. Materials and Methods

### 2.1. Antimicrobial Profile of Lemon Tahiti Essential Oil 

The Tahiti lemon essential oil (Phytoterápica, Rio de Janeiro, Brazil) was extracted from Tahiti lemon peel by cold pressing. Before analysis, the oil was stored in a dark and refrigerated environment (5 °C). The antimicrobial activity of Tahiti lemon essential oil against *Escherichia coli* ATCC 25922 and *Staphylococcus aureus* ATCC 25923 (from American Type Culture Collection (ATCC), Manassas, VA, USA) was screened by the disk diffusion test [22]. For this test, *Escherichia coli* and *Staphylococcus aureus* were activated in a brain–heart infusion broth (Neogen, Lansing, MI, USA) for 24 h at 36 °C. After the incubation period, they were standardized in sterile saline (NaCl 0.85%) until a turbidity compatible with grade 0.5 of the McFarland scale (1.5 × 10^8^ CFU/mL) was obtained. One hundred microliters of the bacterial strains were seeded onto the surface of Mueller–Hinton agar (Kasvi, Paraná, Brazil) Petri dishes. Then, 5 sterile filter paper discs (6 mm; Macherey-Nagel, Düren, Germany) containing, or not, antimicrobial substances were inoculated per dish. Of the 5 inoculated discs, 3 discs contained Tahiti lemon essential oil at a concentration of 1% (*v*/*v*), 1 disc contained 5% dimethyl sulfoxide (negative control; Sigma-Aldrich, Saint Louis, MO, USA), and 1 disc contained 30 µg of cefadroxil (positive control; Laborclin, Paraná, Brazil). The zone of inhibition was measured in a single plane with a digital caliper with 0.001-mm precision (Mitutoyo, Suzano, São Paulo, Brazil) after 24 h of incubation of the plates at 36 °C; then, the results were recorded.

### 2.2. Coating Manufacturing

A method adapted from Sothornvit and Pitak [23] was used for coating preparation. GBF (6% *w*/*w*; Bio Mundo, Brasília, Brazil) and glycerol (50% *w*/*w* of GBF; Dinâmica, São Paulo, Brazil) were dissolved in 400 mL of distilled water. After 30 min of constant stirring at 80 °C, the solution was cooled to 40 °C. Then, 1% (*w*/*w*) Tahiti lemon essential oil was added and stirring continued for another 10 min until completion. Before being added, the essential oil was diluted in Tween 80 (Dinâmica, São Paulo, Brazil) until it reached the desired concentration (1% *v*/*v*). 

### 2.3. Antimicrobial Profile of the Coatings

An antimicrobial analysis of the coatings was also carried out before they were applied to the eggs to verify that they met the antimicrobial criteria. The analysis followed the same protocol as the antimicrobial analysis of the Tahiti lemon essential oil mentioned above. The only difference was that discs containing 1% Tahiti lemon essential oil were replaced with discs containing 20 µL GBF or 20 µL GBF plus Tahiti lemon essential oil.

### 2.4. Eggs, Coating Application, and Egg Storage Conditions

One hundred and sixty-two eggs, with apparently clean and intact shells, were acquired immediately after collection from a quail-rearing system (Planaltina, Federal District, Brazil). The quails were 14 weeks old. No eggs were washed or sanitized. The eggs were randomly divided into three treatments: (1) uncoated eggs, (2) eggs coated with GBF, and (3) eggs coated with GBF/TAH. There were 7 repetitions per treatment (each egg had 1 repetition). The eggs were immersed in the coatings and dried on iron grids at 29.59 ± 0.38 °C and 40.24 ± 0.20% relative humidity. Then, the eggs were placed in sterilized plastic trays and stored for 21 days. From the beginning to the end of the egg storage, the temperature and humidity of the storage environment were monitored every 5 min with a HOBO data logger (Onset Computer Corp., Bourne, MA, USA). 

### 2.5. Assessment of the Internal Quality of Eggs

Seven eggs had their internal quality evaluated weekly in relation to egg weight loss (%, EWL), HU, Yolk index (YI), albumen, and yolk pH (Table 1). 

### 2.6. Microbial Count of Shell and Egg Contents

In total, 9 eggs per treatment were separated immediately after collection and on the 21st day of storage for microbiological analysis of the eggshell, according to the methodology used by Wells et al. [26], with some changes. The shell microbiota was extracted by rinsing, followed by massaging a pool of 3 eggs in 60 mL of 0.1% peptone saline solution inside a sterile bag. Serial dilutions were used only for uncoated egg rinses. One milliliter of the final rinse solution from each treatment repetition was plated on Plate Count Agar (Ionlab, Paraná, Brazil) to count the total aerobic mesophilic bacteria. One milliliter was also plated on Violet Red Bile Glucose Agar (Ionlab, Paraná, Brazil) to count the bacteria of the Enterobacteriaceae family. For both microbial groups, the colonies were counted after incubating the plates at 36 °C for 48 h and were expressed in log_10_ CFU/mL. On the 21st day of storage, the same 9 eggs/treatment used to evaluate the microbiological quality of the shell were used to evaluate the microbiological count of the internal content of the egg, according to a method adapted from Figueiredo et al. [27]. The eggs were immersed for 5 min in 70% alcohol and dried for 30 min at room temperature. Then, the eggs were broken with a sterile stick; the contents of a pool of 3 eggs (18 mL; 6.0 mL of each egg) were extracted and homogenized with 162 mL of 0.1% peptone saline solution. The dilution, plating, incubation, and colony-counting procedures were similar to those performed in the microbiological analysis of eggshells. Both tests were performed in triplicate.

### 2.7. Sensory Analysis of Eggs

On the last day of storage, ten eggs per treatment were separated for sensory analysis, according to a methodology adapted from Nwamo et al. [28]. The analysis involved a panel made up of 10 volunteer judges in a technical course in the field of agricultural sciences, 50% men and 50% women aged between 20 and 40 years. All judges agreed to participate in the analysis by signing the informed consent form. Eggs were cooked at 100 °C for 15 min after boiling. For each treatment, each panel member received a coded whole egg, halved, for an assessment of the color, aroma, odor, texture, taste, and general acceptability using a 9-point hedonic scale; a score of 1 was provided when the panel member “disliked extremely” and a score of 9 was provided when “extremely liked” the egg. Water was supplied directly after the analysis of each treatment.

### 2.8. Statistical Analysis

Means were calculated and reported as mean ± SD. A completely randomized design was used to evaluate parameters of the internal quality (EWL, HU, YI, albumen, and yolk pH), microbiological quality (shell and egg content), and sensory quality of the egg (color, aroma, odor, texture, taste, and general acceptability). PROC GLM from SAS Studio University Edition software (SAS Inst. Inc., Cary, NC, USA) was used to perform an analysis of variance on the data. Significant differences (*p* < 0.05) between the treatment means were tested by Tukey’s test. Non-normal data were compared by the Kruskal–Wallis test using the PROC NPAR1WAY procedure.

## 3. Results and Discussion

### 3.1. Antimicrobial Profile of Tahiti Lemon Essential Oil and Coatings

Tahiti lemon essential oil showed inhibition zones of 9.54 ± 0.72 mm and 10.44 ± 0.91 mm for *E. coli* and *S. aureus*, respectively. Therefore, Tahiti lemon essential oil was added to the GBF coating as an antimicrobial agent. Likewise, the GBF/TAH formulation showed halos of inhibition against these microorganisms (*E. coli*—8.44 ± 0.86 mm and *S. aureus*—9.86 ± 0.64 mm), evidencing the antimicrobial action of the essential oil impregnated in the coating, as the GBF alone did not show antimicrobial activity in vitro. The antimicrobial action of Tahiti lemon essential oil was also demonstrated by Oliveira et al. [20]. Typically, the antimicrobial responses of essential oils are due to their effects on the bacterial system, which can result in combined or non-combined disturbances to that system, including blockages of energy production and active transport [29]. 

### 3.2. Egg Storage Conditions

**Figure 1:**
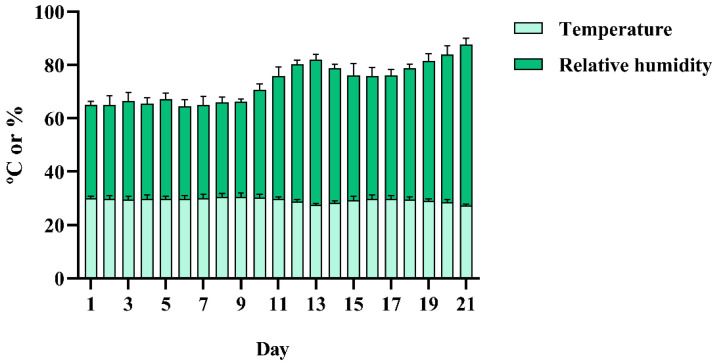
Daily average temperature and relative humidity during 21 days of storage.

Figure 1 shows how the daily temperature and humidity behaved over the 21 days of egg storage. Overall the average temperature and relative humidity were 29.53 ± 1.36 °C and 43.26 ± 8.10%.

### 3.3. Assessment of the Internal Quality of Eggs

The initial internal egg quality (analysis of 15 eggs on day 0) based on HU, YI, albumen, and yolk pH was 93.41 ± 3.95, 0.43 ± 0.05, 8.80 ± 0.14, and 6.33 ± 0.29, in that order.

#### 3.3.1. Egg Weight Loss (EWL)

EWL serves to measure changes in egg life activities [30]. The EWL gradually increased during storage, presenting similarly (*p* = 0.3973) at 21 days among uncoated eggs (9.98 ± 2.29%), eggs coated with GBF (9.18 ± 1.60%), and eggs coated with GBF/TAH (7.93 ± 1.93%). However, considering the average loss observed between 7 and 21 days of storage, GBF/TAH coated eggs (5.87 ± 2.17%) showed lower EWL (*p* = 0.0072) compared to uncoated eggs (7.18 ± 2.99%) and eggs coated with GBF (6.98 ± 2.55%) (Table 2). This result is supported by Oliveira et al. [20], who reported that the combination of cassava starch and Tahiti lemon, ginger, or lemongrass essential oil also significantly reduced EWL after 35 days of storage at 20 °C. Coatings formulated with hydrophilic and hydrophobic substances positively aggregate their mechanical and barrier properties [31]. Thus, the addition of Tahiti lemon essential oil to GBF may have improved its barrier properties against H_2_O evaporation, as the essential oil may have strengthened the hydrophobic fraction of the hydrophilic coating [32]. Essential oils can promote emulsion stability and facilitate the formation of egg coatings with good compatibility of hydrogen bonding interactions, optimizing their barrier properties [33].

#### 3.3.2. Haugh Unit (HU)

**Figure 2:**
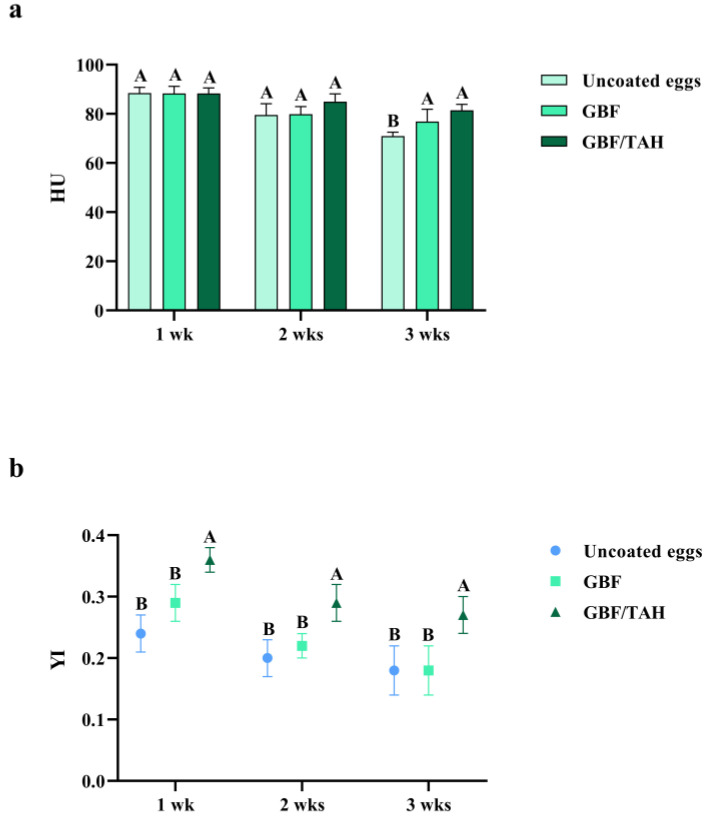
Effect of treatments on (**a**) Haugh unit (HU) and (**b**) yolk index (YI) during 3 weeks of storage at room temperature. ^A,B^ Means with different uppercase letters differ significantly (*p* < 0.05). GBF, green banana flour; GBF/TAH, green banana flour plus Tahiti lemon essential oil. Egg quality score based on HU: AA, excellent (≥72); A, high quality (71–60); B, average quality (59–31); and C, low quality (≤30) [36].

The HU of the eggs tended to decrease with advancing storage time, with the lowest mean (*p* = 0.0009) observed in the third week for uncoated eggs (70.94 ± 1.63, grade A), followed by eggs coated with GBF (76.90 ± 4.94; grade AA) and GBF/TAH (81.47 ± 2.38, grade AA) (Figure 2a). Considering the average loss observed between 7 and 21 days of storage, the GBF/TAH coating also maintained (*p* < 0.0001) the highest HU (84.93 ± 3.79, AA), while no significant differences were reported in GBF-coated eggs and uncoated eggs (Table 2). The reduction in albumen quality is the main reason for the reduction in HU. Albumen quality decreases, particularly at room temperature, due to the depletion of albumen protein content and protein–protein interactions, making the thick gelatinous albumen more liquefied [34]. This disturbance in the albumen protein system appears to be minimized when eggs are coated with GBF/TAH, ensuring better albumen quality. Sun et al. [35] observed that the HU of eggs coated with chitosan plus beeswax plus basil essential oil (C-BW-BEO) slowly decreased over a storage period, reaching day 35 with significantly higher values (classified as grades AA) than those of uncoated eggs. Song et al. [33] reported that a coating associating chitosan, shellac, and pine needle essential oil (CS-PNEO) was beneficial for the HU of eggs stored for 28 days at 25 °C, showing significantly higher values than those of uncoated eggs. Both studies showed that essential oils strengthen the effects of hydrophilic coatings on preserving eggs. This can be useful for the egg industry and retail outlets that have difficulties in providing a cooling system for eggs.

#### 3.3.3. Yolk Index (YI)

After coating with GBF/TAH, the YI of the eggs reduced more slowly (*p* < 0.0001; reaching 0.27 ± 0.03 in the 3rd week) than the eggs coated with GBF (0.18 ± 0.04) and the uncoated eggs (0.18 ± 0.04) (Figure 2b). Considering observations of the storage between 7 and 21 days, eggs coated with GBF/TAH continued to show better YI (*p* < 0.0001; 0.31 ± 0.04) compared to those using the other treatments (Table 2). The loss of quality of the yolk seems to depend on the loss of quality of the albumen since the irreversible changes that occur in the yolk, such as the fragility of the vitelline membranes and liquefaction, result from the diffusion of water that occurs due to changes in the protein system of the albumen [37]. Therefore, the YI-based yolk quality was expected to be better for eggs coated with GBF/TAH, as it contributed to smaller changes in albumen quality, as supported by the higher HU. Farnejad et al. [38] showed that *Satureja hortensis* and *Satureja mutica* essential oils added to chickpea protein isolate coating positively influenced the yolk quality of eggs stored for 7 weeks at 20 °C.

#### 3.3.4. Albumen and Yolk pH

The albumen and yolk pH tended to increase over the 21 days of storage; however, weekly, there was no significant difference (*p* = 0.6985; *p* = 0.4510, respectively) between eggs coated with GBF, eggs coated with GBF/TAH, and eggs that were uncoated. However, the albumen and yolk pH observed between 7 and 21 days of storage were lower (*p* = 0.0004; *p* = 0.0107) for eggs coated with GBF/TAH (8.83 ± 1.06; 6.53 ± 1.20) than for eggs coated with GBF (9.04 ± 1.07; 6.73 ± 1.18) and uncoated eggs (9.05 ± 0.98; 6.78 ± 1.22), respectively (Table 2). The lower pH of the albumen for GBF/TAH probably occurred because it hindered the passage of CO_2_ from the inside to the outside of the egg, which may have minimized the negative effects on the balance of the carbonate–bicarbonate buffer system. This system, when unbalanced, together with the loss of CO_2_, favors an increase in the pH of the albumen [39]. The lower pH of the yolk may be a reflection of the slow penetration of albumen water molecules into the yolk, as the transition of water at the albumen-yolk interface is the main justification for the increase in yolk pH [40]. These results reinforce the significant role that Tahiti lemon essential oil played as a coating additive for eggs in preserving the albumen and yolk quality. Studies have also confirmed lower albumen and yolk pH values on the last day of storage for eggs coated with coatings containing essential oils [20,33,41]. 

### 3.4. Microbial Count of Shell and Egg Contents

The microbiota of an egg can be attributed to the oviduct, wind, nest, and egg collection and must be monitored periodically so that the burden on the egg’s lifespan, human health, and the economy is minimal. GBF/TAH-coating reduced (*p* < 0.0001) the total count of aerobic mesophilic bacteria in eggshells and egg contents on the 21st day of storage (0.43 ± 0.23 log_10_ CFU/mL, 0.86 ± 0.16 log_10_ CFU/mL, respectively); this is especially evident when compared to other treatments (Table 3). 

On day 0, GBF/TAH reduced bacteria counts in the eggs it was applied to; however, they did not differ substantially from the uncoated eggs. However, GBF/TAH maintained low bacterial levels in the shell throughout storage, achieving the lowest load and the best microbiological quality of the eggshell on day 21. This finding may be an explanation for the lower bacterial count in the content of eggs coated with GBF/TAH. The more microorganisms that survive on the eggshell, the more easily they will penetrate the egg contents [42]. Thus, GBF/TAH may have hindered the survival of microorganisms in the shell, reducing the chance of them penetrating the eggs. The antimicrobial behavior of GBF/TAH was expected, as Tahiti lemon essential oil showed in vitro antimicrobial activity against *Escherichia coli* and *Staphylococcus aureus* at 1%, before and after being combined with GBF coating. These microorganisms are inserted within the group of mesophilic bacteria. Tahiti lemon essential oil has a residual antimicrobial characteristic that can last for a significant period [20]. This may have been one of the main reasons why the GBF/TAH coating made the eggshell less favorable for the survival and infiltration of microorganisms through the pores. These results corroborate with those of Eddin and Tahergorabi [43], who reported that the level of *Salmonella enterica serovar* Typhimurium in the shells of eggs coated with sweet potato starch plus 6% thyme essential oil was below the detectable level in the 1st and 5th weeks of storage at 25 °C. In another study, pectin and gum arabic coatings plus carvacrol and eugenol were able to significantly minimize the presence of *Salmonella enterica serovar* Enteritidis in eggshells [18]. In the present study, there was no growth of the bacteria of the Enterobacteriaceae family on the shells or in the contents of the eggs.

### 3.5. Sensory Analysis of Eggs

On the 21st day of storage, eggs from different treatments showed similar sensory profiles (*p* > 0.05) based on color, aroma, odor, texture, flavor, and general acceptability (Table 4). Thus, the tested coatings did not change the typical characteristics of the eggs, which is a big step for the GBF/TAH coating to become a strong candidate for industrial application, as consumer acceptability is one of the most important factors in marketing a product. Furthermore, it appears that the GBF-based coating had some undesirable effects of the essential oil imperceptible, including its recognizably strong aroma. Similarly, Caner and Cansiz [44] reported that consumers were unable to differentiate eggs with or without chitosan coating; comparatively, they had the same general acceptability.

## 4. Conclusions

The application of Tahiti lemon essential oil to the GBF coating is an effective blending approach to reduce the microbial load of the shell and egg contents and preserve the sensory and internal quality of the eggs. Therefore, the application of this coating can be useful to reducing refrigeration costs and the strong side effects of high temperatures on egg viability.

## Figures and Tables

**Table 1:** Parameters used to evaluate the internal quality of eggs during storage for 21 days at room temperature.

Parameters	Evaluation
EWL	The initial egg weight (IEW) and the final egg weight (FEW) were measured to calculate the EWL, which is EWL = (IEW − FEW)/IEW × 100.
HU	Albumen height (H) and egg weight (W) were measured to calculate the HU, where HU = 100 log (H + 7.57 – 1.7 W^0.37^) [24].
YI	The height (h) and diameter (d) of the yolk were measured to calculate the YI, where YI = h/d [25].
Albumen and yolk pH	Albumen and yolk pH were measured individually using a calibrated digital pH meter (206–pH2, Testo, Lenzkirch, Baden-Württemberg, Germany) after the previous analyses.

**Table 2:** Effect of treatments on egg weight loss (EWL), Haugh unit (HU), yolk index (YI), albumen, and yolk pH between days 7 and 21 of storage at room temperature.

Treatment	EWL (%)	HU	YI	Albumen pH	Yolk pH
Uncoated eggs	7.18 ± 2.99 ^a^	79.65 ± 7.89 ^b^	0.20 ± 0.04 ^c^	9.05 ± 0.98 ^a^	6.78 ± 1.22 ^a^
GBF	6.98 ± 2.55 ^a^	81.68 ± 6.09 ^b^	0.23 ± 0.05 ^b^	9.04 ± 1.07 ^a^	6.73 ± 1.18 ^a^
GBF/TAH	5.87 ± 2.17 ^b^	84.93 ± 3.79 ^a^	0.31 ± 0.04 ^a^	8.83 ± 1.06 ^b^	6.53 ± 1.20 ^b^
*p* value	0.0072	<0.0001	<0.0001	0.0004	0.0107

^a, b, c^ Means with different lowercase letters in the columns (treatment effect considering the entire storage period) differ significantly (*p* < 0.05). GBF, green banana flour; GBF/TAH, green banana flour plus Tahiti lemon essential oil.

**Table 3:** Counts of total aerobic mesophilic bacteria on eggshell surfaces of the different treatments at 0 and 21 days of storage and in the egg content at 21 days of storage at room temperature.

Treatment	Total Aerobic Mesophilic Bacteria Count (log_10_ CFU/mL)		
	Day 0 (Eggshell)	Day 21 (Eggshell)	Day 21 (Content)
Uncoated eggs	1.87 ± 0.54 ^A,ab^	1.95 ± 0.07 ^A,a^	1.90 ± 0.09 ^a^
GBF	1.93 ± 0.16 ^A,a^	1.67 ± 0.23 ^A,a^	1.52 ± 0.22 ^a^
GBF/TAH	0.95 ± 0.57 ^A,b^	0.43 ± 0.23 ^A,b^	0.86 ± 0.16 ^b^
*p* value	<0.0001		0.0007

^a,b^ Means with different lowercase letters (column; treatment effect within each day of storage) differ significantly (*p* < 0.05); ^A^ means with the same uppercase letters (row; effect of day of storage within each treatment) do not differ significantly (*p* > 0.05). GBF, green banana flour; GBF/TAH, green banana flour plus Tahiti lemon essential oil.

**Table 4:** Effect of treatments on sensory characteristics of eggs stored for 21 days at room temperature.

Sensory Characteristics	Treatment			
	Uncoated Eggs	GBF	GBF/TAH	*p* Value
Color	7.50 ± 1.65 ^a^	7.10 ± 1.66 ^a^	7.40 ± 1.65 ^a^	0.8542
Aroma	7.40 ± 2.10 ^a^	7.00 ± 2.26 ^a^	7.60 ± 1.51 ^a^	0.7948
Odor	8.50 ± 0.97 ^a^	8.50 ± 0.53 ^a^	8.30 ± 1.06 ^a^	0.8440
Texture	7.80 ± 1.55 ^a^	7.80 ± 0.63 ^a^	8.00 ± 1.15 ^a^	0.9081
Taste	7.50 ± 1.18 ^a^	7.10 ± 1.52 ^a^	7.30 ± 1.70 ^a^	0.8350
General acceptability	7.50 ± 2.12 ^a^	7.20 ± 1.40 ^a^	7.40 ± 1.17 ^a^	0.9148

^a^ Means with the same lowercase letters in the row do not differ significantly (*p* > 0.05). GBF, green banana flour; GBF/TAH, green banana flour plus Tahiti lemon essential oil.

## Data Availability

Not applicable.
